# From Psychological Skills Training to Observable Behavior: A Pilot Study Operationalizing the 5Cs as a Behavioral Coding Framework for Youth Central Defenders

**DOI:** 10.3390/bs16091472

**Published:** 2026-08-24

**Authors:** Daði Rafnsson, Peter O’Donoghue, Chris Harwood, Karl Steptoe, Berglind Sveinbjörnsdóttir, Hafrún Kristjánsdóttir

**Affiliations:** 1 Department of Psychology, Reykjavik University, 102 Reykjavik, Iceland; berglindsv@ru.is; 2 Department of Sport Science, Reykjavik University, 102 Reykjavik, Iceland; petero@ru.is (P.O.); hafrunkr@ru.is (H.K.); 3 School of Science and Technology, Nottingham Trent University, Nottingham NG11 8NS, UK; chris.harwood@ntu.ac.uk; 4 School of Sport, Exercise and Health Sciences, Loughborough University, Loughborough LE11 3TU, UK; k.j.steptoe@lboro.ac.uk

**Keywords:** football, performance analysis, 5Cs, observation, behavior analysis

## Abstract

Observing psychological skills in football can inform coaching feedback but is methodologically challenging. Meaningful behaviors can be difficult to isolate from tactical and technical actions due to the game’s complexity. This pilot study asked whether Harwood’s 5Cs framework (commitment, communication, concentration, control, confidence) could be operationalized into a behavioral coding system for central defenders, with what intra-rater consistency a single expert observer could apply it, and what patterns emerged across pre-, mid-, and post-intervention phases. Two youth teams (U15, U17) from a Major League Soccer Development Academy participated. Video footage from 16 competitive matches was analyzed using a template derived from co-designed 5Cs role descriptions. Five central defenders appearing in all three phases were retained for exploratory descriptive analysis. Event-detection agreement was 73.3%; mean intra-operator κ among matched events was 0.917 for behavior type and 0.799 for outcome, falling to 0.427 (95% CI 0.300–0.554) when detection and classification errors were combined. This indicates moderate overall agreement, consistent with single-expert feasibility rather than general reliability. The sample (*n* = 5) was not powered for inferential comparisons. Patterns are reported descriptively as rates per 90 min, success percentages, and within-player trajectories. The study offers a transparent template for operationalizing the 5Cs as observable behaviors, a prototype rather than a validated instrument.

## 1. Introduction

Observing and analyzing psychological skills (PS) in football presents a considerable methodological challenge. The sport functions as a complex sociotechnical system, in which player actions are highly context-dependent and rarely meaningful when examined in isolation (McLean et al., 2017; Salmon & McLean, 2020). Even seemingly straightforward behaviors can be difficult to code reliably across observers or analytical systems, complicating the development of consistent observational frameworks (Robinson & O’Donoghue, 2009).

Despite these challenges, observational methods have increasingly been used to analyze the psychological and behavioral aspects of football performance. By structuring observation through coding or category systems, behavioral performance data can be quantified (Preciado et al., 2019) and capture meaning from spontaneous behavior in competitive and training contexts without relying on self-reports or retrospective recall (Barreira et al., 2020). Prior research has employed such approaches to study penalty shootouts (Jordet et al., 2007; Wood et al., 2015; Furley et al., 2020), visual scanning behaviors (Jordet et al., 2020; Pokolm et al., 2022), and non-verbal communication (Halldórsson, 2018; Lian et al., 2026). Findings from case studies (Halldórsson, 2018), women’s professional leagues (Lian et al., 2026), and matches played during the COVID-19 pandemic era without spectators (Leitner & Richlan, 2021) further demonstrate that psychological and communicative behaviors can be systematically observed and analyzed within competitive football contexts. By identifying behavioral patterns, observational results can inform coaching and performance interventions to create training situations relevant to the competitive tasks ahead (Maneiro et al., 2021).

Despite the increased adoption of sport psychology in football, coaches often struggle to operationalize abstract psychological concepts (Corsie et al., 2024). To enhance their understanding, clarity in operational definitions is required (Ashdown et al., 2026). For this purpose, psychological role descriptions for each playing position were recently co-designed with professional coaches (Rafnsson et al., 2026). These descriptions illustrate how the 5Cs framework (C. Harwood, 2008) for psychological skills training (PST) in football can facilitate the construction of observable target behaviors on the pitch. It is important to distinguish three things that can all occur in a single passage of play. One is the technical-tactical execution of an action (e.g., stepping up to compress space), the observable tactical action this produces at the team level (e.g., a coordinated defensive line), and the psychological behavior the action is taken to express (e.g., communication and concentration). The 5Cs framework does not treat these dimensions as separate or competing categories. Rather, it foregrounds the psychological qualities that underpin tactically and technically meaningful actions. The coding system evaluated here, therefore, captures behaviors that are also tactically meaningful, but interprets them through the lens of the psychological skills they are taken to express. For example, central defenders were expected to demonstrate commitment by physically engaging (touching) the opponent when competing for the ball, communicate effectively by giving clear verbal and non-verbal directions, and maintain concentration by scanning and anticipating play. The role descriptions provide the operational definitions of each position, allowing coaches to both teach and assess PS in competitive contexts. This operationalization provided a practical basis for both intervention and performance analysis through direct observation in this study.

An effective PST intervention should be structured, progressive, developmentally appropriate, and embedded within everyday coaching practice (Camiré & Trudel, 2014; Diment, 2014; Thrower et al., 2024). The 5Cs model of positive youth development in football (C. Harwood & Anderson, 2015) was chosen for this study as it is one of the most widely applied frameworks for PST in youth football. The 5Cs are commitment, communication, concentration, control, and confidence, providing athletes, coaches, and support staff with a vehicle into sport psychology and a shared language for PS. The 5Cs have been adopted in youth academy environments to guide assessment (Steptoe et al., 2016), shape coaching philosophy, and integrate psycho-social skills into daily practice (Steptoe et al., 2019). They have served as a foundation for youth development programs (C. Harwood, 2008), a behavioral assessment tool for players, a framework to support parental involvement (Kramers et al., 2023), and a reflective tool for coaches (Wadsworth et al., 2020). Its theory-based construction, adaptability across various contexts, and accessibility for applied use make it a highly suitable psychological skills training (PST) model for academy football.

Previous evaluations of PST interventions based on the 5Cs model have primarily relied on self-assessment questionnaires or post-intervention interviews (C. Harwood, 2008; C. G. Harwood et al., 2015). This reliance on self-report measures reflects a broader trend in football-based PST research, in which effectiveness is typically measured through participants’ perceptions or psychological test scores rather than observable performance outcomes (Thelwell et al., 2006; Olmedilla et al., 2019). Most existing PST interventions have targeted specific psychological skills, such as mindfulness (Trajkovski & Newland, 2022) or motivation (Barnicle & Burton, 2016), that align well with available questionnaire instruments. However, it remains unclear whether improvements in self-reported PS or positive intervention experiences translate into enhanced competitive performance on the field.

Recent advances in video analysis, player-tracking technologies, and automated performance systems have transformed how coaches and researchers examine football, influencing both player development and recruitment practices (Rein & Memmert, 2016; Pires & Santos, 2018; Goes et al., 2021; Frevel et al., 2022). Supported by these technologies, expert observers can integrate contextual knowledge to interpret subtle tactical and psychological dynamics, particularly when guided by structured analytical frameworks (Grosprêtre & Gabriel, 2021; Mommers et al., 2023). Also, noting how traditional performance analysis runs the risk of reductionist approaches, O’Sullivan et al. (2025) have encouraged coaches and practitioners to adapt ecological dynamics and systemic theories to inform their practice. This approach transforms the detached analyst into an embedded relational facilitator who co-creates common understanding within teams through dialogue.

The authors of this research have considerable applied experience in football through coaching, sport psychology services, and performance analysis. Our approach is pragmatic, using research methods that should be realistically helpful in applied, real-life situations. The present pilot study, therefore, addresses the two following research questions:

1. To what extent can the 5Cs framework be operationalized into a behavioral coding system for central defenders, and with what level of intra-rater consistency by an expert observer?
2. In a pilot application of the coding system, what preliminary behavioral patterns are observed across pre-, mid-, and post-intervention phases following a structured 5Cs intervention?

In line with the design’s exploratory nature, the former question is treated as the study’s primary contribution. The second question is addressed using descriptive summaries and individual player trajectories, with the understanding that the small analytic sample limits statistical power and prevents inferential conclusions about intervention effects.

## 2. Materials and Methods

### 2.1. Participants

Two teams from a Major League Soccer (MLS) Development Academy participated in the intervention: 26 players (ages 13 to 14 at time of intervention and observation) on the U15 team and 28 players (ages 15 to 16) on the U17 team. A high standard of play was prioritized to minimize the influence of technical or tactical limitations on the observations. The MLS is the top-tier men’s professional football league in the United States, with academies designed to provide pathways for young players into professional careers (Major League Soccer, 2010). From those teams, eight central defenders were selected for observation between September and November 2022. Four of the observed players were members of the U17 team (aged 15–16 years), and four were members of the U15 team (aged 14–15 years; no 13-year-old player played as a central defender). The analysis included eight games from each team, sixteen in total. For the final analysis, only players who appeared in matches across all three phases (pre-, mid-, and post-intervention) were retained, resulting in five players (three U17 and two U15). All had been selected to represent the club’s academy from lower-ranked teams in the area. They came in for training five days a week and typically played a competitive game, either home or away, on the weekends. Biological maturity was not assessed as the included players were 14–16 years old, inter-individual differences were not accounted for, and biological maturity is considered an alternative explanation for behavioral change in Section 4 and Section 4.1. The coaches were not told which positions or players would be analyzed, and they selected their teams and substitutions based on tactical and developmental reasons. Central defenders were chosen for analysis as they are less frequently rotated during matches than players in other positions. Table 1 below describes the players included in the final analysis. During the observation period, three more players appeared in the Central defender position across the two teams. Two only appeared in one game, and one in three games, but only in two phases.

### 2.2. Procedure

#### Intervention Design and Implementation

The intervention was based on the 5Cs framework (C. Harwood, 2008), with the aim of introducing PS and establishing a shared language around key concepts within the club. The program was delivered to all the players by the first author in collaboration with the club’s High-Performance Specialist (HPS). The U15 team began its program two weeks before the U17 team’s due to academy scheduling and the ability to adjust the sessions to the teams’ training plans. All classroom sessions were delivered as planned. There were 4 sessions per team, 45–55 min each, using identical materials and structure for both teams. Attendance was mandatory for all players, and no absence was recorded. Each session focused on one ‘C’ (with concentration and control taught together due to their close interconnection). Sessions typically began with the HPS sharing a personal story related to the focal C, followed by a senior player role model who exemplified the concept. Players then discussed personal experiences, before the researcher introduced relevant theory and video examples. Reflection activities included journaling about how the C related to players’ own experiences, and each session concluded with a ‘call to action’ to apply the C in upcoming games. Posters featuring the senior role model’s reflections were displayed in the locker room after each session. Upon completion of the classroom phase, players received written descriptions of the 5Cs roles to clarify the key behaviors. The head coaches were responsible for delivering the concepts during daily training, with the first author present to provide support, feedback, and suggestions before, during, and after training.

Head coaches were responsible for embedding the 5Cs into daily training, with support from the primary researcher, who provided feedback and planning assistance. Integration was guided by the PROGRESS model (C. Harwood & Anderson, 2015), which encourages coaches to promote the 5Cs in practice, role-model desired behaviors, give players ownership, foster skill growth, reinforce positive behaviors, empower peer support, and facilitate self-review. Coaches were asked to integrate the session themes into training sessions. Integration was supported through pre-session briefings, first with the head coach, then with the team. The researcher additionally had daily informal check-ins with the coaches (in person or by phone). Session progress was monitored through the first author’s presence at training, video recordings, voice recordings, and journal entries. Implementation fidelity was not formally quantified, and this is addressed in Section 4.1. A detailed account of the intervention and coaches’ perceptions is reported separately (Rafnsson et al., in press). The present study draws on the same intervention period but is distinct in focus and does not duplicate the analysis reported there. Its concern is only the development and pilot application of the behavioral coding framework for central defenders and the behavioral observation data.

### 2.3. Data Collection

Data were collected between September and November 2022. The subsequent period has been used for analysis, framework refinement, intra-rater assessment, and methodological integration with the broader PST program reported in Rafnsson et al. (in press). Each team played eight matches during this period, giving a total of 16 observations. Match duration was 90 min for the U17 team and 80 min for the U15 team. The schedule comprised three pre-intervention matches, three during the four-week PST program, and two in the two weeks following the program. The first author coded all footage and was aware of the phase (pre-, mid-, or post-intervention) to which each match belonged. No blinding was therefore possible, making confirmation bias possible. Matches were recorded either manually with a camera device on a tripod or automatically using Veo cameras, depending on what was available at each venue. Both captured the play from the middle sideline in a television-style format, following the ball, but the systems differed in their fields of view and tracking behavior. Tripod footage occasionally lost sight of central defenders when the team attacked high up the pitch. At the same time, Veo’s wide-angle capture retained them more consistently, though with fewer close-ups available. The club provided the research team with unedited MP4 files via Dropbox. Audio quality was generally poor due to environmental conditions, such as wind or rain, as well as equipment limitations regarding audio recordings, making consistent analysis of verbal communication impractical. As noted in Section 4.1, variability in recording conditions can limit the comparability of observations across matches. However, it was important for the research to test the method in applied, real-life situations that coaches and clubs face daily.

**Measurements**

The behavior coding template was derived using 5C role descriptions for central defenders developed in a collaborative study with expert coaches (Rafnsson et al., 2026) and refined through a five-month pilot study using video footage from 10 National Collegiate Athletic Association (NCAA) women’s soccer matches. During testing, the items that proved difficult to observe reliably were removed, mostly related to verbal communication due to unreliable sound recording. The reasons for inclusion or exclusion at different stages are described in Table 2 below.

The final tagging guide is presented in Table A1 as Appendix A and includes tagging descriptions with the assigned C, original role description, examples of successful and unsuccessful actions, and decision rules.

Three properties of the coding system should be distinguished and are not claimed to the same degree. Content validity concerns whether the behavioral indicators adequately represent the 5Cs constructs. The indicators were co-designed with professional coaches (Rafnsson et al., 2026), which supports content relevance but is not equivalent to formal validation. Ecological validity concerns whether the behaviors are meaningful in real competitive play, and coding live match footage supports this. Six player performances from three matches (one in each phase) were used in the intra-operator agreement study. The performances included 12 behavior types that were coded during the study. These performances were by three U17 players (Players 1–3), lasting in total 450 min, which is 19.1% of the video-recorded data used in the study. The number of events differed between the first and second observations of each performance. The matching process resulted in 566 events during the six performances, of which 331 were events recorded during both observations, but not always agreeing on the behavior type and outcome. Events from the two observations of each performance were matched manually by the second author, using a single spreadsheet with one row per event. Matching was based primarily on the recorded time of the event, within a tolerance window of 5 s, and secondarily on behavior type and, to a lesser extent, outcome; where more than one candidate event fell within the window, the closest match on these criteria was taken. Where an event in either observation could not be matched with any event in the other, “BLANK” was recorded for the behavior type and outcome of the missing event, so that every detection disagreement was retained in the dataset. There were 35 (10.6%) matched events for which the recorded times differed by more than 5 s; these arise because some behaviors occur over several seconds and can legitimately be tagged at different moments in the two observations. Because behavior type and outcome partly informed the matching, the classification agreement reported below is conditional on the matched events and may be higher than it would be under matching on time alone. We therefore also report agreement in which detection and classification errors are combined, treating events identified in only one observation as disagreements: for behavior classification, mean P_0_ = 0.545 (95% CI 0.445–0.645) and mean κ = 0.427 (95% CI 0.300–0.554), determined across the six performances using the t distribution. Event-detection agreement was calculated per observation as the number of matched events divided by the number of events recorded in that observation, giving twelve values (two per performance). The mean was 73.3% (95% CI 67.9–78.8%), with the confidence interval determined from the twelve values using the t-distribution (t = 2.201, df = 11). The pooled Dice coefficient, 2M/(n1 + n2) = 2 × 331/897, is 73.8%. Intra-operator agreement for the classification of behavior type and outcome was calculated using the 331 matched events from the six performances to avoid double-counting event detection errors.

The coaches wanted their central defenders to engage opponents physically and compete bravely for the ball. These were labeled as commitment behaviors. The communication behaviors were to remain calm and directive, use clear, direct verbal and nonverbal messages, and hold oneself and others accountable in the event of pressure or mistakes. The central defender was expected to display concentration by avoiding changing positions with the other central defender, scanning the field in anticipation, being first to balls played through or high, and directing the team to keep its shape. Control involved stepping up or down with the other defenders and organizing the defensive line when a player left it. Finally, they were expected to show confidence by staying on their feet and only sliding in emergencies, avoiding high-risk passes when the stakes were high, and passing decisively at all times to minimize risky turnovers.

### 2.4. Reflexivity and Researcher Position

The first author delivered the classroom component of the intervention, supported the coaches’ embedding of the 5Cs in daily training, and subsequently conducted all behavioral coding. This dual role introduces a clear risk of confirmation bias. A researcher who has invested in the intervention may, consciously or otherwise, be inclined to code ambiguous instances in ways that favor the expected direction of change. We acknowledge this risk directly and describe the steps taken to mitigate it below. First, all coding decisions were governed by the written operational definitions and decision rules presented in Table A1 (Appendix A), which were finalized during a five-month pilot phase using NCAA women’s soccer footage prior to data collection on MLS academy teams. The coding guide, including the handling of ambiguous cases, was fixed before any intervention match was coded. Second, intra-rater reliability was established by recoding where the first author did not review the original codes. The re-coding was entered blind into a separate file, with comparison to the original conducted by the second author only after both were complete. Reliability was calculated across behavior identification and success-of-behavior dimensions. Third, the first author kept a reflective research journal across the coding period, in which coding decisions, ambiguous cases, and emerging observations were recorded. These entries were discussed in supervision meetings throughout the analysis phase. Fourth, we foreground the methodological consequences of the single-coder design in Section 4.1 and treat the absence of inter-rater reliability assessment as the most significant methodological limitation of the study. Finally, this exploratory research was motivated by piloting an approach to performance analysis that has potential benefits for practitioners who develop fluency in both the demands of football coaching and the language and application of the 5Cs. The first author’s relevant expertise includes a Union of European Football Associations (UEFA) A coaching license, over 20 years of football coaching experience across international contexts, more than five years of applied use of performance analysis software, and more than six years of applied engagement with the 5Cs framework in club and research settings. The results presented in this study should be interpreted as evidence of single-expert feasibility but not as evidence of the framework’s reliability in general, which would require independent inter-rater assessment. The agreement estimates reported below quantify the degree of consistency achieved by one experienced observer.

### 2.5. Data Analysis

Video data was coded using Nacsport Basic Plus (Nacsport, Gran Canaria, Spain) with a custom tagging window. Behaviors were tagged as they occurred and classified as successful or unsuccessful. Each clip captured a 14 s sequence (seven seconds before and after the tagged behavior) to ensure the full action was observed. Coding was highly time-intensive, and analyzing a single player in a 90 min match required approximately 150–180 min. Through The jamovi project (2024) statistical analysis tool, Cohen’s Kappa (κ) was calculated to assess intra-observer reliability.

The first author conducted all coding under supervision from the second and fifth authors. Observational notes and reflections were also recorded in a research journal. Analysis began with the number of successful and unsuccessful instances for each behavior and was then scaled up to 90 min using Equation (1) for consistency and to account for substitutions. The percentage success of a behavior within a performance was determined using Equation (2). For the analysis, players were included only if they had performance data from all three periods (pre-, mid-, and post) during the data collection, which took place before, during, and after the administration of the PST program. Therefore, two players were removed, one from each team, as they only made one appearance at central defender during the data collection period. An additional player from the U15 team (Player 2 U15) was removed as he did not appear in the post-intervention phase. This left five players (three U17, two U15) for whom a weighted average was calculated for each period, ensuring that frequencies from 5 min would not equal those from 35 min; thus, the player was the unit of analysis. Equation (3) was used to determine the weighted average for each frequency per 90 min and the percentage success variable for each player in each phase. Then the data were repackaged so that the pre-, mid- and post-intervention data were side by side for analysis. This way, averages of one value for instances occurring in each period were produced. This prevents the occurrence of low frequencies, and, as expected, some variables may not function properly because the behavior occurs infrequently or not at all. At this point, behaviors that had not occurred in one or more periods had to be eliminated, leaving eight for the eventual report. This was originally based on fifteen behaviors from the role description and twelve that were included in the data collection procedure.

Frequency per 90 min, F90 = Frequency of behavior × 90/min played(1)

Percentage success for a behavior, PS = 100 × S/(S + F)(2)

where S is the number of successful applications of a behavior, and F is the number of unsuccessful applications of the behavior

(3) $$\text{Phase-specific}\;\mathrm{weighted}\;\mathrm{average},\;\mathrm{FW}\;=\;\frac{\sum_{i=1}^{K}\left(f_{i}\times t_{i}\right)}{\sum_{i=1}^{K}t_{i}}$$

where K is the number of matches played in the given phase, f_i_ is the frequency of the given behavior per 90 min in match i, and t_i_ is the number of minutes played in match i.

### 2.6. Ethical Considerations

The study was conducted in accordance with the Declaration of Helsinki (World Medical Association, 2013). Data were collected in 2022 under the participating club’s institutional authorization, and a guardian-consent process (described below). Prior to 2025, the universities in Iceland did not have their own research ethics committees, and the national bioethics committee only addressed research in the health and medical sectors. Reykjavik University’s research ethics committee was established in 2025. Formal ethics approval from the Reykjavik University ethics committee (approval no. 2026003, granted 2 February 2026) was obtained retrospectively, prior to the analysis reported here. The approval explicitly covered the retrospective analysis and publication of the 2022 dataset. It covers the use of video recordings involving minors, the guardian consent and club authorization procedures used in 2022, and the absence of written participant assent. We confirm that, at the time of collection in 2022, the recording and use of footage involving minors complied with the club’s institutional requirements and applicable legal requirements, based on guardian consent and club authorization. The latter university approval was sought to meet the requirements for academic publication.

Because all participants were minors, written informed consent was obtained in 2022 from each player’s legal guardian on their behalf via an online form that covered participation, the recording and analytical use of match video, and potential academic publication. Consent was approved by the guardians of all 26 players on the U15 team and all 28 players on the U17 team before 25 October 2022. Written assent was not collected from the players themselves. Players were informed of what participation entailed, including the option to withdraw at any time. Formal assent was not obtained. Players were informed of what participation entailed through email, an online meeting with their guardians, and an introduction at the club, which included the option to withdraw at any time. The absence of written participant assent is acknowledged as a limitation, reviewed as part of the 2026 ethics approval. One U17 player left the club after his guardians signed the consent form, but he had not yet been able to take part. The club’s Senior Director of Sports Medicine and High Performance authorized the club’s participation and the use of game footage, documented by email, and the head coaches consented to being recorded. He also secured approval from the club’s human resources and legal departments, as well as its academy director, by the end of June 2022. Video files were recorded and stored by the club’s performance department and securely shared with the first author via encrypted transfer. The files were stored in secure university servers, and access was restricted to the research team and used solely for the purposes described here.

## 3. Results

Table 3 summarizes the intra-operator agreement for the six performances that were included in the intra-operator agreement study. For each of the six performances, a confusion matrix was constructed, and P_0_ and κ were calculated. The values reported are the arithmetic means of the six performance-specific estimates, and 95% confidence intervals were determined from the six values using the t distribution (t = 2.571, df = 5). For behavior type, mean P_0_ was 0.941 (95% CI 0.927–0.955) and mean κ was 0.917 (95% CI 0.891–0.943); for outcome, mean P_0_ was 0.921 (95% CI 0.880–0.962) and mean κ was 0.799 (95% CI 0.678–0.920). However, the level of intra-operator agreement is moderate, with a 73.3% event-detection agreement. Table 4 shows that the majority of disagreements were detection errors and that these have an impact on most behavior types. Table 5 shows the agreements and disagreements when classifying the outcome of the 331 matched events. Kappa calculated directly from the pooled outcome matrix in Table 5 is 0.839; the mean of the performance-specific values is 0.799, and we report the latter, with the performance as the unit of analysis.

**Figure 1:**
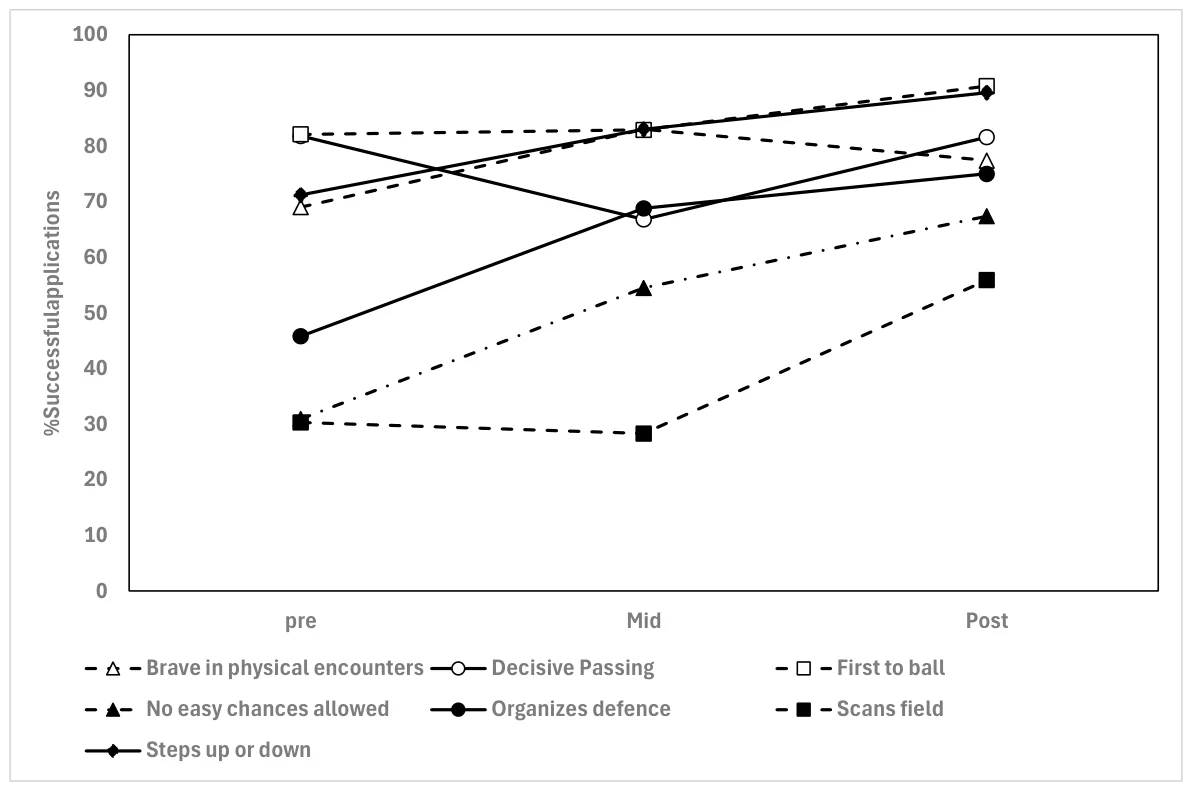
Group-level trajectories of behavior success percentages across phases (aggregated across the five players). Note: individual-player trajectories appear in Appendix B, Figure A1.

Descriptive statistics for behavior frequencies and success percentages across the three phases are presented in Table 6 and Table 7, and individual trajectories are shown in Figure 1. Evidence of change between phases was not tested using inferential statistics.

Among the success percentages, *No easy chances* allowed rose progressively from pre- to post-intervention at the group level as seen below in Table 7.

This pattern was also visible in individual trajectories (Figure 1). *Steps up or down*, and *Decisive passing* showed modest group-level declines. Meanwhile, the remaining behaviors were broadly stable. Two behaviors, *Calm and decisive non-verbal communication* and *Organizes defense*, could not be summarized as success percentages because they had zero frequencies at one or more phases for one or more players. These zero frequencies are themselves informative about how rarely these behaviors were observed.

**Figure A1:**
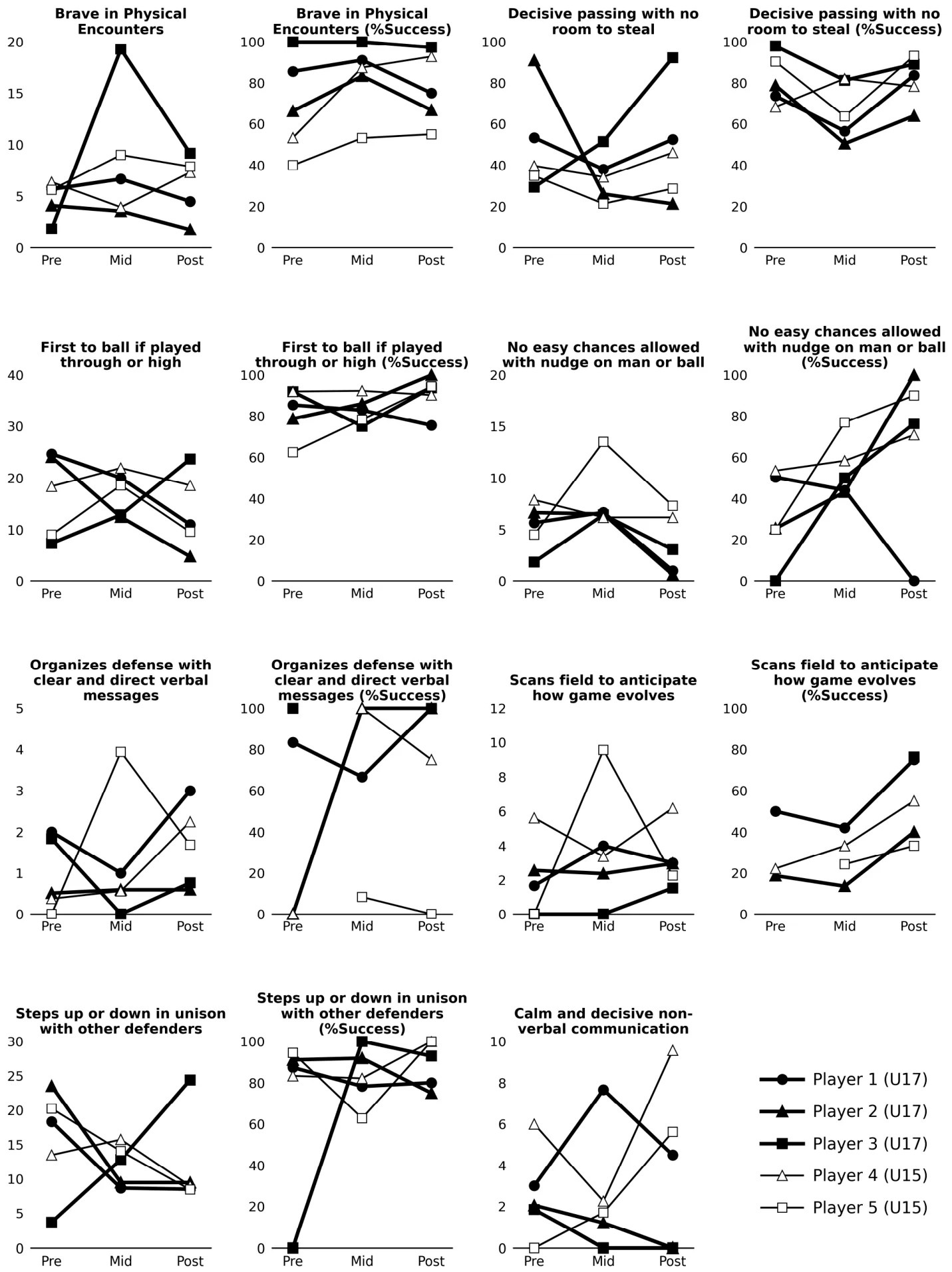
Frequency per 90 min and percentage success of behaviors by different players.

Additionally, frequencies per 90 min and percentage success of behaviors by different players are presented as Figure A1 in Appendix B. The underlying per-player, per-phase counts, exposure minutes, and derived values are provided in Table A2 in Appendix C.

## 4. Discussion

This study aimed to examine the extent to which the 5Cs framework can be operationalized as a behavioral coding system for central defenders, and to quantify the intra-rater consistency with which an expert observer could apply it. Moreover, in a pilot application of the coding system, what preliminary behavioral patterns are observed across pre-, mid-, and post-intervention phases following a structured 5Cs intervention?

The findings extend prior work on PST in football by moving beyond self-report evaluations toward direct observation and behavioral assessment. Earlier 5Cs interventions (C. Harwood, 2008; C. G. Harwood et al., 2015) documented perceived improvements in players’ commitment, communication, and control, but relied on post-session questionnaires and interviews. Other PST programs have measured constructs such as motivation (Barnicle & Burton, 2016) or mindfulness (Trajkovski & Newland, 2022) using psychometric instruments that capture perception rather than performance. The current study contributes a complementary perspective by examining whether those psychological qualities manifest in observable, position-specific actions during competition. In this respect, it aligns with calls within applied sport psychology to connect internal processes with externally verifiable behaviors (Diment, 2014; C. Harwood & Anderson, 2015). The findings also correspond with a broader trend in football research that integrates technological and contextual performance analysis (Rein & Memmert, 2016; Pires & Santos, 2018; Goes et al., 2021; Frevel et al., 2022) as well as moving away from a mechanist reductionist approach to an embedded, relational one (O’Sullivan et al., 2025). Through systematic video tagging and contextual interpretation, expert observers can capture psychological expressions that traditional statistics overlook (Grosprêtre & Gabriel, 2021; Mommers et al., 2023). The present study illustrates how combining these analytic advances with a theoretically grounded psychological model can yield richer insights into player development. Although limited by sample size, combining sport psychology frameworks and performance analysis techniques offers a promising approach to a more holistic evaluation of player behavior and its impact on subsequent individual development strategies.

A central contribution lies in the study’s methodological innovation: the operationalization of the 5Cs framework into observable, role-specific behavioral indicators suitable for systematic coding. The use of position-specific descriptors enabled psychological constructs to be directly linked to the functional demands of the central defender role. This responds to persistent critiques that PST often lacks ecological validity and practical measurability (Corsie et al., 2024). By developing and pilot-testing behavioral categories derived from coaches’ co-designed role descriptions (Rafnsson et al., 2026), the study provided an applied template that other analysts and coaches can adapt.

The framework was applied with 73.3% event-detection agreement (95% CI 67.9–78.8%) and, among matched events, mean intra-operator κ of 0.917 (95% CI 0.891–0.943) for behavior type and 0.799 (95% CI 0.678–0.920) for outcome; with detection and classification errors combined, κ was 0.427 (95% CI 0.300–0.554). We take this to indicate a substantive finding about the complexity of the task as much as a limitation of the method. In a dynamic sport such as football, deciding whether a given action counts as an instance of a 5Cs behavior, rather than as an adjacent tactical action, or as background play, is the genuinely difficult part of applied psychological coding. That difficulty is consistent with previous observational work on complex psychological behaviors in football (Halldórsson, 2018; Pokolm et al., 2022; Lian et al., 2026). We interpret the findings here cautiously, as they indicate that the behavior counts reported here carry real measurement uncertainty, and that the patterns here would be materially strengthened by inter-rater replication with a second independent coder. We nonetheless judge the framework to be feasible for exploratory applied use and refinement, rather than robust for confirmatory measurement, at this stage of development.

During the intervention period, several behaviors were observed more frequently than at baseline in this small sample, spanning commitment (bravery in 1v1s), control (organizing and directing teammates), and concentration (anticipatory scanning). These categories map onto C. Harwood’s (2008) conceptualization of the 5Cs as observable and coachable aspects of psychological performance, and the patterns are consistent with the kind of incremental behavioral adaptation described in youth development contexts (Camiré & Trudel, 2014; Thrower et al., 2024). We emphasize, however, that the design cannot attribute these patterns to the intervention, and we therefore describe them as behavioral patterns observed during the intervention period rather than as evidence of skill acquisition or intervention uptake. The observed patterns, therefore, align with the notion of progressive psychosocial skill development embedded within daily coaching routines (Thrower et al., 2024). These are descriptions of observed behavioral trajectories using 5Cs-informed behavioral indicators, not evidence of change in underlying psychological constructs. Whether such behavioral patterns reflect psychological development is a question for adequately powered designs with independent coding.

The mean frequency of *Organizes defense* and the success percentage of *No easy chances* allowed were higher during the intervention phase than at baseline and remained above baseline post-intervention. These within-player descriptive patterns cannot be attributed to the intervention. While the direction of some changes is not inconsistent with early engagement with the 5Cs framing, the design cannot demonstrate this, and the observed patterns cannot be attributed to the intervention within a 12-week window among academy footballers aged 13–16 without considering alternative explanations. Players in this age group are undergoing maturation, which could affect some of the observed shifts, such as physical bravery or recovery speed. Also, the coaching staff may have adjusted their tactical approaches during the data-collection period for reasons unrelated to the 5Cs intervention.

Furthermore, opponent quality varied across the eight matches per team, and some of the variability in defensive behavior likely reflects opponent characteristics. Finally, coaches’ feedback and continuous development, which are common in an academy setting, may have contributed to behavioral change independently of the 5Cs intervention. The study was not designed to discriminate among these possibilities, so we present the behavioral patterns as consistent with, but not causally attributable to, the intervention.

### 4.1. Limitations

This study was designed as a pragmatic, context-based pilot, and several of its limitations follow from that intent rather than from oversight. Because of the complex, situated nature of analyzing psychological skills in competition, testing, or achieving inter-rater reliability was not the primary goal of the research. The approach is based on the authors’ combined experience in coaching, sport psychology, and performance analysis within real academy settings. Our aim was therefore to build on our work as applied practitioners and academics to pilot a pragmatic solution for analyzing psychological skills that practitioners could use in environments far from laboratories or other controlled settings.

In academy football, variability can be introduced by several things, such as weather, opponents, travel, team selection, and video recording conditions. Traditional performance analysis research strives for replicability and low variance, and we acknowledge that standard. So far, however, the analysis of psychological skills and performance has tended to run aground when these skills are treated as though they were passes or shots. Those actions are comparatively simple to observe yet are hardly ever perfectly codable themselves. We therefore offer a context-based pilot that could encourage others to consider operationalizing behaviors, rather than validated instruments. The reliability sample covered 19.1% of the coded dataset, drawn from three matches (one per phase) involving only U17 players, and several behaviors contributed few events to the agreement estimates. So, the agreement estimates may not generalize across all behaviors and phases. The event matching in the agreement study was performed by a co-author who was not blinded to the recorded classifications, and matching was partly informed by behavior type and outcome; the classification agreement estimates are therefore conditional on the matching procedure. That said, the absence of inter-rater assessment does mean the agreement reported here is intra-rater only. It establishes consistency within a single expert observer but cannot show whether an independent coder would identify the same behaviors. Thus, the behavior counts should be read as observer-specific rather than framework-general. Independent inter-rater evaluation remains the natural next step for anyone wishing to build on this work.

Several further features of the design constrain how far the observed patterns can be interpreted, and many reflect the applied conditions in which the framework is meant to operate. There was no control or comparison group, so behavioral change cannot be separated from secular trends, normal maturation, or concurrent coaching. Fidelity was not formally quantified, but both teams received the same number of classroom and training sessions, and the classroom sessions used the same teaching material and structure. There was no blinding, and the coder was aware of each match’s phase, which, combined with the dual role, leaves a residual risk of confirmation bias that intra-rater agreement cannot detect. For behaviors without a bounded opportunity set, opportunity identification relied on the coder’s judgment, guided by the decision rules, making the success denominators partly observer-dependent. Biological maturity was not assessed, although the players aged 13–16 were in a period of rapid maturation that could itself shift behaviors such as physical bravery or recovery speed. There was no follow-up beyond the two post-intervention weeks, so it is unknown whether any patterns consolidate or fade. The two-week start dates between the U15 and U17 groups meant the phase windows did not coincide in calendar time. Recording conditions varied between Veo and tripod or handheld footage, limiting comparability across matches. Several of these sources of variability, such as opponent quality, selection, scheduling, and recording, are characteristic of competitive academy football rather than artifacts that a field study can fully remove. They are part of what makes psychological-skills observation in this setting demanding.

The analytic sample is very small, with five players, each contributing two to three matches per phase. The study is therefore a pilot, and no inferential comparisons were conducted. The descriptive patterns reported can not support conclusions in either direction about intervention effects. Behaviors were excluded at two stages. During the pilot, items that could not be reliably observed (largely verbal communication owing to poor audio), and, post hoc, behaviors with zero frequency in one or more phases. The latter introduces a risk of post hoc selection that a longer observation window might have mitigated. Finally, the role descriptions underlying the coding guide were co-designed with coaches and refined through this study, but have not been independently validated. The framework is offered as an adaptable, context-sensitive prototype rather than as an instrument assuming a single objective ground truth for psychological performance. Our approach, asking coaches which behaviors they consider important in each position and then mapping these onto the 5Cs framework to sharpen clarity and provide a common language, is one we find genuinely promising. Our central motivation for presenting this study is to encourage others to take up and build on these ideas in their own contexts.

### 4.2. Future Research

Assessing inter-rater reliability with a second independent coder within a club, using its own role descriptions, would be valuable for further developing this method. Longitudinal designs with larger, more diverse samples would be useful for increasing statistical power and providing stronger indications of intervention effects. Comparative studies across genders, cultures, and developmental stages would help clarify how the 5Cs operate in different contexts. Further work should examine the framework’s potential applications in player recruitment and talent identification, as well as its role in player development. Studies conducted over a full season may reveal whether the observed behavioral trends persist over time or are related to performance outcomes, such as match results, coach ratings, or selection decisions. Additionally, as automated tagging and computer vision systems continue to improve, there is potential to explore semi-automated coding approaches that could reduce observer burden and enable broader use of the framework in applied settings. As a prototype, the framework’s most immediate research need is independent inter-rater validation within a club, using its own role descriptions, before any claims about measurement properties or intervention effects can be supported. Written assent was not obtained from players, although they were informed of the study and their right to withdraw. Future work should include formal assent procedures.

### 4.3. Practical Applications

In youth academy contexts, recruiting large samples across equivalent positions is an impractical endeavor, and small samples as presented here cannot support inferential claims. Thus, transparent reporting provides a more honest picture of behavioral variation across phases than *p*-values.

We suggest three concrete applications: The first is embedding 5Cs concepts within everyday video analysis, which could help align the language of coaches, players, and sport psychologists, creating a shared reference for psychosocial feedback. The second is using the observational guide developed here as a developmental monitoring tool through structured video review. And the third is making abstract psychological constructs more tangible for athletes through reflective debriefing meetings that foster discussions about on-field behaviors. This integrative approach complements the growing demand for psychologically informed environments within elite youth development systems (Thrower et al., 2024). It aligns with contemporary sport-organization models that emphasize holistic player development.

## 5. Conclusions

The integration of theory-driven behavioral indicators with performance analysis has the potential to bridge the gap between sport psychology and applied coaching practice. This exploratory study evaluates a behavioral coding framework grounded in the 5Cs, applied to central defenders in competitive youth football. A single expert coder applied the framework with moderate event-detection agreement and high intra-operator agreement when classifying the type and outcome of matched events. With detection and classification errors combined, overall agreement was moderate. Preliminary behavioral patterns were observed across pre-, mid-, and post-intervention phases. These patterns cannot be interpreted causally, given the sample size and design, and warrant further investigation with larger samples and inter-rater designs. At this stage, the framework should be considered a prototype for applied development rather than a validated observational instrument. Its primary contribution is the coding framework itself, presented here with operational definitions, decision rules, and worked examples, which offers coaches, analysts, and sport psychologists a transferable template for operationalizing 5Cs language in video review and developmental monitoring. The next priority for this framework should be an inter-rater reliability evaluation with an independent coder, followed by studies with larger samples across full competitive seasons.

## Figures and Tables

**Table 1:** Central defenders included in the final data analysis.

Player	Age	Category	Matches				Minutes Played			
			Phase 1	Phase 2	Phase 3	Total	Phase 1	Phase 2	Phase 3	Total
Player 1	16	U17	3	3	2	8	270	270	180	720
Player 2	16	U17	3	2	2	7	176	152	152	480
Player 3	15	U17	2	1	2	5	49	28	118	195
Player 4	14	U15	3	2	2	7	240	160	160	560
Player 5	14	U15	1	2	2	5	80	160	160	400

**Table 2:** Role descriptions retained or excluded from final analysis.

Role Description (5C Domain)	Pilot Coding	Study Coding	Reliability Sample	Final Analysis	Exclusion Reason
Bravely enters physical encounters without hesitation. (Commitment)	Yes	Yes	Yes	Yes	—
Does not allow easy chances, by always getting a nudge on the ball or man. (Commitment)	Yes	Yes	Yes	Yes	—
Calm and decisive non-verbal communication at all times. (Communication)	Yes	Yes	Yes	Yes	—
Organizes defense through clear and direct verbal and non-verbal messages. (Communication)	Yes	Yes	Yes	Yes	—
Maintains calm and directive attitude towards teammates. (Communication)	Yes	Yes	Yes	No	Excluded after observation: lacking data from all phases
Holds self and others accountable when under pressure or mistakes happen. (Communication)	Yes	Yes	Yes	No	Excluded after observation: could not be observed reliably (inconsistent audio). Too infrequent.
Avoids crossing lines with other central defender, passing the attacker on, and holding the line. (Communication)	Yes	No	No	No	Excluded after pilot: unreliable to observe (inconsistent audio)
Constantly scans field to anticipate how game evolves. (Concentration)	Yes	Yes	Yes	Yes	—
Anticipates how game evolves by being first to ball if played through or high. (Concentration)	Yes	Yes	Yes	Yes	—
Anticipates how game evolves by directing team up the field to keep shape in attack. (Concentration)	Yes	No	No	No	Excluded after pilot: indistinguishable from Steps up or down (inconsistent audio)
Steps up or falls back in unison with other defenders. (Control)	Yes	Yes	Yes	Yes	—
Organizes defense line to tighten if a player leaves it. (Control)	Yes	Yes	Yes	No	Excluded after observation: lacking data from all phases
Stays on feet at all possible times, only slides in emergencies. (Control)	Yes	Yes	Yes	No	Excluded after observation: lacking data from all phases
Does not try high-risk passes or moves when stakes are high. (Confidence)	Yes	No	No	No	Excluded after pilot: “high-stakes” too variable to operationalize.
Decisive passing that does not leave opponents room to steal. (Confidence)	Yes	Yes	Yes	Yes	—

**Table 3:** Summary of detection and intra-operator agreement statistics.

Match	Player	Minutes	Event Detection			Behavior Classification		Outcome Classification	
(Phase)			Observation 1	Observation 2	Matched	P_0_	k	P_0_	k
1 (Pre)	1	90	112	128	85	0.929	0.908	0.894	0.691
4 (Mid)	1	90	102	101	79	0.962	0.950	0.975	0.940
4 (Mid)	2	90	69	63	42	0.929	0.885	0.881	0.683
7 (Post)	1	28	27	21	16	0.938	0.925	0.938	0.862
7 (Post)	2	62	68	63	51	0.941	0.903	0.941	0.866
7 (Post)	3	90	76	67	58	0.948	0.929	0.897	0.752

P_0_ is the proportion of agreements; k is the kappa statistic.

**Table 4:** Confusion matrix for behavior classification within six performances.

Observation 1	Observation 2													
	Brave in Physical Encounters	Calm and Decisive Non-Verbal Communication	Decisive Passing with No Room to Steal	First to Ball If Played Through or High	Holds Self and Others Accountable	Maintain Calm Towards Teammates	No Easy Chances Allowed with Nudge on Man or Ball	Organizes Defense	Scans Field to Anticipate How Game Evolves	Stays on Feet	Steps Up or Down in Unison with Other Defenders	Tightens Defensive Line	No Behavior Recorded	Total
Brave in physical encounters	31	0	0	1	0	0	0	0	0	0	0	0	8	40
Calm and decisive non-verbal communication	1	5	1	0	0	0	0	1	0	0	0	0	9	17
Decisive passing with no room to steal	1	0	157	2	0	0	1	0	0	0	0	0	28	189
First to ball if played through or high	1	0	2	59	0	0	1	0	1	0	2	0	14	80
Holds self and others accountable	0	0	0	0	0	0	0	0	0	0	0	0	1	1
Maintain calm towards teammates	0	0	0	0	0	2	0	0	0	0	0	0	1	3
No easy chances allowed with nudge on man or ball	0	0	0	1	0	0	10	0	0	0	0	0	8	19
Organizes defense	0	0	0	0	0	0	0	3	0	0	0	0	3	6
Scans field to anticipate how game evolves	0	0	0	1	0	0	0	0	4	0	2	0	14	21
Stays on feet	0	0	0	0	0	0	0	0	0	0	0	0	2	2
Steps up or down in unison with other defenders	0	0	0	0	0	0	0	0	0	0	41	0	35	76
Tightens defensive line	0	0	0	0	0	0	0	0	0	0	0	0	0	0
No behavior recorded	10	13	22	14	0	1	6	7	13	0	25	1	0	112
Total	44	18	182	78	0	3	18	11	18	0	70	1	122	566

**Table 5:** Confusion matrix for outcome classification within six performances.

Observation 1	Observation 2		
	Successful	Unsuccessful	Total
Successful	178	13	191
Unsuccessful	13	127	140
Total	191	140	331

**Table 6:** Behavior frequency per 90 min by phase (mean ± SD across players).

Variable	Phases		
	Pre	Mid	Post
Brave in physical encounters	4.7 ± 1.8	8.5 ± 6.4	6.1 ± 3.0
Calm and decisive non-verbal communication	2.6 ± 2.2	2.6 ± 3.0	3.9 ± 4.1
Decisive Passing	49.7 ± 24.8	34.2 ± 11.6	48.2 ± 27.7
First to ball	16.7 ± 8.2	17.2 ± 4.3	13.5 ± 7.5
No easy chances allowed	5.3 ± 2.3	7.9 ± 3.2	3.6 ± 3.0
Organizes defense	0.9 ± 0.9	1.2 ± 1.6	1.7 ± 1.0
Scans field	2.0 ± 2.3	3.9 ± 3.5	3.2 ± 1.8
Steps up or down	15.9 ± 7.7	12.2 ± 3.0	12.0 ± 7.0

**Table 7:** Success percentage by phase (mean ± SD across players).

Variable	Phases		
	Pre	Mid	Post
Brave in physical encounters	69.0 ± 24.1	83.0 ± 17.8	77.4 ± 17.7
Calm and decisive non-verbal communication	NA	NA	NA
Decisive Passing	81.8 ± 12	66.8 ± 14.4	81.6 ± 11.4
First to ball	82.1 ± 12.2	82.9 ± 6.7	90.8 ± 9.3
No easy chances allowed	30.7 ± 21.9	54.5 ± 13.9	67.4 ± 39.4
Organizes defense	45.8 ± 53.4 ^#^	68.8 ± 43.2 ^#^	75.0 ± 43.3
Scans field	30.3 ± 17.1 ^$^	28.3 ± 12.2 ^#^	55.9 ± 19.6
Steps up or down	71.2 ± 40.0	83.0 ± 14.1	89.6 ± 11.5

Note: NA = no instances observed for one or more players in that phase. ^#^ Mean + SD were calculated from four players with contributing values. ^$^ Mean + SD were calculated from three players with contributing values.

## Data Availability

Only de-identified, coded data may be shared upon request to the first author and subject to ethical and institutional restrictions. Video recordings involving minors, and any notes or data that could identify the players, cannot be shared.

## Abbreviations

The following abbreviations are used in this manuscript:

PS	Psychological skills
PST	Psychological skills training
MLS	Major League Soccer
HPS	High Performance Specialist
PROGRESS	Promote, Role Model, Ownership, Growth, Reinforce, Empower, Self-Review
NCAA	National Collegiate Athletics Association
UEFA	Union of European Football Associations

## Appendix A

**Table A1:** The 5Cs tagging guide with worked examples and decision rules.

Tagging Description	Role Description	Successful Action	Unsuccessful Action	Decision Rules
Brave in physical encounters (Commitment)	Bravely enters physical encounters without hesitation	A long ball is played into the central defender’s zone, and a striker positions his body in front to receive and hold it up. The defender steps in, makes deliberate contact to unbalance the striker as the ball arrives, and gets a touch on it to knock it clear to a team-mate or out of play.	In a similar duel just outside the box, the defender hesitates half a step before committing, makes contact late, and the striker holds him off with his upper body, manages to escape the challenge and keep the ball himself or pass to a teammate.	Code only when the central defender initiates or sustains physical contact with an opponent in contesting the ball. If contact is incidental check under First to ball if applicable, or do not code. A successful instance requires that the defender’s bravery influenced the outcome of the duel.
No easy chances allowed (Commitment)	Does not allow easy chances, by always getting a nudge on the ball or man	A cross is delivered low into the box with an attacker arriving at pace. The central defender reacts to get a touch on the ball a split second earlier to win, block or deflect it rather than letting the attacker meet it cleanly.	A cutback from the byline finds an attacker unmarked at the penalty spot. The central defender stays put and does not close the gap or attempt a block as the attacker strikes, and the shot goes unchallenged.	Code only when the play occurs in or around the defensive penalty area with a clear scoring opportunity at stake. If the same action occurs higher up the pitch where no scoring opportunity is at stake, code under First to ball instead. If both No easy chances allowed and Brave in physical encounters could apply, No easy chances allowed takes precedence when a scoring chance is the focus; otherwise Brave in physical encounters applies.
Maintain calm towards teammates (Communication)	Maintains calm and directive attitude towards teammates.	During and after events of high importance (opposition chances, set-pieces, teammate mistakes), the central defender should maintain composure and engage in calm verbal and non-verbal communication with teammates.	In similar highly important situations, the central defender becomes visibly agitated and loses composure with negative effects on verbal and non-verbal communication with teammates.	Code if noticing obvious verbal or non-verbal communication during or after highly important situations. The key is to maintain composed communication. Being loud and animated is fine, as long as the communication is directive, with a problem-solving focus.
Organizes defense (Communication)	Organizes defense through clear and direct verbal and non-verbal messages.	The central defender makes gestures and/or verbally gives instructions to other players when they leave their position so that they can work together to compress space and pick up players who might exploit the space.	In similar situations the central defender does not offer instructions through verbal or non-verbal gestures. The area of the field that the player has vacated is exploited by the opposition moving into space to receive and progress with the ball.	Code when gesture is observed and directed at own teammates (pointing, open-hand directions, deliberate eye contact, body orientation toward a teammate). Do not code routine waving or unfocused arm movement.
Holds self and others accountable (Communication	Holds self and others accountable when under pressure or mistakes happen.	When the defense is under pressure or mistakes happen, the central defender must verbally and non-verbally demand accountability of self and others.	When a high-pressure moment has been relieved and/or if a mistake has happened, the central defender must verbally and non-verbally admit own responsibility or demand of others to maintain focus and accept theirs.	Code when player displays non-verbal and verbal acceptance of own responsibility after a mistake or a dangerous moment has been avoided. Also code if the player demands of others to maintain focus and perform responsibly.
Calm and decisive non-verbal communication (Communication)	Calm and decisive non-verbal communication at all times.	The opposition is in possession wide and the defensive line is adjusting laterally. The central defender makes clearly visible gestures directing the full-back to push up toward a late-arriving midfielder to indicate a pick-up.	During the same kind of shift with the ball on the opposite flank, the central defender keeps his eyes fixed on the ball, offering no visible gesture. A team-mate is left uncertain about whether to track a runner and ends up reacting late.	Code when the gesture is observable as directed at a specific teammate or area of the pitch (pointing, open-hand directions, deliberate eye contact, body orientation toward a teammate). Do not code routine waving or unfocused arm movement. If the gesture’s purpose is to organize the defensive line in response to a positional change, code under Organizes defense instead.
Scans field (Concentration)	Constantly scans field to anticipate how game evolves before and opportunity to receive or engage the ball.	With possession being built up by his own goalkeeper, the central defender makes obvious head-checks over each shoulder in the seconds before receiving the ball, taking in the positions of attacking opponents and available team-mates.	In a similar build-up moment, the central defender keeps his eyes fixed on the ball at the goalkeeper’s feet without turning his head.	Code when the defender turns their head deliberately to a position outside their immediate line of sight (over shoulder, or away from ball) before the ball arrives to them or a defensive action. A glance without a head turn is not coded. A successful instance requires that the scan occurred early enough and a head turn after the ball has arrived is coded as unsuccessful.
First to ball (Concentration)	Anticipates how game evolves by being first to ball if played through or high	A long diagonal ball is played over the top into the space between the central defender and the full-back. The defender begins his recovery run before the opposing forward reacts and arrives at the ball ahead to gain possession or clear it out of play.	A midfielder’s pass falls between the two central defenders. The defender in closest proximity hesitates before committing, and the opposing forward runs through the gap to collect it first.	Code when two or more players are in genuine contention for the ball and the central defender’s anticipation determines who arrives first. If played directly to the defender with no opponent contesting, do not code. If the action also qualifies under No easy chances allowed (a clear scoring opportunity in or around the defensive box), code there instead.
Steps up or down (Control)	Steps up or falls back in unison with other defenders	With the ball at the opposing full-back’s feet with no immediate forward threat, the defensive partners step up five metres to compress the space. The central defenders move in unison, keeping the line straight to limit space towards the midfielders and increasing chance of offside behind them.	In the same scenario, the defensive partners step up but the central defender hesitates and remains deeper. The line breaks, offering an opportunity to play behind it without being offside.	Code when the defender adjusts their position in coordinated relation to the rest of the defensive line in response to a change in possession, ball location, or opponent positioning. A solo positional adjustment is not coded. If the adjustment is accompanied by a verbal or non-verbal direction to teammates to follow, code that gesture also separately under Organizes defense as they can occur simultaneously.
Tightens defensive line (Control)	Organizes a defensive line to tighten if a player leaves it	The full-back steps out of the defensive line to press an opponent. The central defender immediately calls and/or gestures toward the remaining back line, directing them to slide across and tighten the gap. Within three seconds the line has realigned into a compact shape.	The full-back steps out in the same way, but the central defender gives no verbal or visible instruction to the remaining defenders. The gap is not closed within three seconds.	Code when the defender’s gesture or call is directed at teammates and is intended to produce a coordinated change in defensive shape. A general encouragement or a routine call without a specific organizing function is not coded. If the gesture also serves as non-verbal communication of intention but its primary purpose is to direct defensive shape, code under Tighten defensive line.
Stay on feet (Control)	Stays on feet at all possible times, only slides in emergencies.	The central defender is in the last defensive line to goal and an attacker is running at him with the ball, looking for passing or shooting opportunity. Must stay on feet to maintain body between ball and goal and not slide.	In a comparable action, the central defender is in the last defensive line to goal, and an attacker is running at him with the ball. He decides to slide, allowing the player to move past him, or create a shooting opportunity on goal.	Code on whether the defender remains standing and maintains his body between the ball and the goal. If he slides as a last resort and has a physical encounter, code under Brave in physical encounters.
Decisive passing (Confidence)	Decisive passing that does not leave opponents room to steal the ball	Under moderate pressure from an oncoming player, the central defender plays a firm pass to the defensive midfielder. The weight of the pass is strong enough that a covering opponent cannot step in to intercept it, and the midfielder receives cleanly.	In a comparable action, the central defender plays a soft pass toward the defensive midfielder. An intercepting opponent reads the pass and steps across to win the ball before it arrives.	Code only on the weight of the pass, if it was played appropriately firmly enough that an opponent could not step in to intercept. A firmly weighted pass that is intercepted is coded as unsuccessful but a soft pass that allows the teammate to receive without being intercepted is coded as successful. Direction is irrelevant to this category.

## Appendix C

**Table A2:** Player and phase data.

Behavior/Variable	Player 1			Player 2			Player 3			Player 4			Player 5		
	1	2	3	1	2	3	1	2	3	1	2	3	1	2	3
Minutes	270	270	180	176	152	152	49	28	118	240	160	160	80	160	160
Brave in physical encounters															
Successful events	14	18	8	5	5	2	1	6	11	10	6	12	2	9	8
Unsuccessful events	3	2	1	3	1	1	0	0	1	7	1	1	3	7	6
Total opportunities	17	20	9	8	6	3	1	6	12	17	7	13	5	16	14
Frequency/90 min (wt)	5.7	6.7	4.5	4.1	3.6	1.8	1.8	19.3	9.2	6.4	3.9	7.3	5.6	9.0	7.9
Percentage success (wt)	85.6	91.1	75.0	66.2	83.3 ^#^	66.7 ^#^	100.0 ^#^	100.0	97.4	53.3	87.5	92.9	40.0	53.2	55.0
Calm and decisive non-verbal communication															
Total opportunities	9	23	9	4	2	0	1	0	0	16	4	17	0	3	10
Frequency/90 min (wt)	3.0	7.7	4.5	2.0	1.2	0.0	1.8	0.0	0.0	6.0	2.3	9.6	0.0	1.7	5.6
Decisive Passing															
Successful events	118	70	99	148	23	23	15	13	107	77	50	64	28	25	48
Unsuccessful events	42	44	6	30	21	13	1	3	14	29	11	18	3	13	3
Total opportunities	160	114	105	178	44	36	16	16	121	106	61	82	31	38	51
Frequency/90 min (wt)	53.3	38.0	52.5	91.0	26.1	21.3	29.4	51.4	92.3	39.8	34.3	46.1	34.9	21.4	28.7
Percentage success (wt)	73.5	56.5	83.7	78.7	50.4	63.9	98.0	81.3	89.1	68.2	82.1	78.2	90.3	63.6	93.2
First to ball															
Successful events	63	50	18	37	18	8	3	3	29	45	36	30	5	26	16
Unsuccessful events	11	10	4	10	3	0	1	1	2	4	3	3	3	7	1
Total opportunities	74	60	22	47	21	8	4	4	31	49	39	33	8	33	17
Frequency/90 min (wt)	24.7	20.0	11.0	24.0	12.4	4.7	7.3	12.9	23.6	18.4	21.9	18.6	9.0	18.6	9.6
Percentage success (wt)	85.4	82.8	75.4	78.7	86.0	100.0	91.8	75.0	93.8	92.0	92.3	90.2	62.5	78.3	94.4
No easy chances allowed															
Successful events	8	9	0	2	4	1	0	1	1	12	6	8	1	19	12
Unsuccessful events	9	11	2	11	7	0	1	1	3	9	5	3	3	5	1
Total opportunities	17	20	2	13	11	1	1	2	4	21	11	11	4	24	13
Frequency/90 min (wt)	5.7	6.7	1.0	6.6	6.5	0.6	1.8	6.4	3.1	7.9	6.2	6.2	4.5	13.5	7.3
Percentage success (wt)	50.4	44.1	0 ^#^	25.6	43.2	100.0 ^#^	0.0 ^#^	50.0	76.3	53.6	58.3	70.8	25.0	76.9	90.0
Organizes defense															
Successful events	5	2	6	0	1	1	1	0	1	0	1	3	0	1	0
Unsuccessful events	1	1	0	1	0	0	0	0	0	1	0	1	0	6	3
Total opportunities	6	3	6	1	1	1	1	0	1	1	1	4	0	7	3
Frequency/90 min (wt)	2.0	1.0	3.0	0.5	0.6	0.6	1.8	0.0	0.8	0.4	0.6	2.3	0.0	3.9	1.7
Percentage success (wt)	83.3 ^#^	66.7	100.0	0.0 ^#^	100.0 ^#^	100.0 ^#^	100.0 ^#^		100.0 ^#^	0.0 ^#^	100.0 ^#^	75.0 ^#^		8.3	0.0
Scans field															
Successful events	1	5	5	1	1	2	0	0	1	4	2	6	0	4	2
Unsuccessful events	4	7	1	4	3	3	0	0	1	11	4	5	0	13	2
Total opportunities	5	12	6	5	4	5	0	0	2	15	6	11	0	17	4
Frequency/90 min (wt)	1.7	4.0	3.0	2.6	2.4	3.0	0.0	0.0	1.5	5.6	3.4	6.2	0.0	9.6	2.3
Percentage success (wt)	50.0 ^#^	42.1	75.0	18.8	13.6	40.0 ^#^			76.3	22.2	33.3	55.0		24.3	33.3
Steps up or down															
Successful events	44	21	15	37	14	12	0	4	26	31	23	16	17	16	15
Unsuccessful events	11	5	2	9	2	4	2	0	6	5	5	0	1	9	0
Total opportunities	55	26	17	46	16	16	2	4	32	36	28	16	18	25	15
Frequency/90 min (wt)	18.3	8.7	8.5	23.5	9.5	9.5	3.7	12.9	24.4	13.5	15.8	9.0	20.3	14.1	8.4
Percentage success (wt)	87.4	78.3	80.0	91.1 ^#^	91.8	75.0 ^#^	0.0 ^#^	100.0	92.9	83.3	82.1	100.0	94.4	62.8	100.0

(wt) These are weighted averages to account for differing match lengths. ^#^ The percentage success was determined from a subset of matches within the phase where the frequencies were greater than 0. Note: For Calm and decisive non-verbal communication, occurrences were recorded without a success/failure classification; success percentages are therefore not defined.
